# Distal Transradial Access in Anatomical Snuffbox for Coronary Angiography and Intervention: An Updated Meta-Analysis

**DOI:** 10.1155/2021/7099044

**Published:** 2021-07-22

**Authors:** Chendi Liang, Qinghua Han, Yongping Jia, Chunyu Fan, Gang Qin

**Affiliations:** ^1^Shanxi Medical University, Taiyuan, Shanxi, China; ^2^Department of Cardiology, The First Hospital of Shanxi Medical University, Taiyuan, Shanxi, China

## Abstract

**Objective:**

The previous meta-analysis has assessed that distal transradial access (dTRA) in anatomical snuffbox is safe and effective for coronary angiography and intervention and can reduce radial artery occlusion. However, since the publication of the previous meta-analysis, several observational studies have been added, so we performed an updated meta-analysis to include more eligible studies to compare distal transradial access in anatomical snuffbox with conventional transradial access (cTRA).

**Method:**

Pubmed, Embase, and Cochrane Library databases were searched for relevant studies from the literature published until 5 January 2021 to evaluate catheterization/puncture failure, hematoma, radial artery spasm, radial artery occlusion (RAO), access time, fluoroscopy time, radiation dose area product, total procedure time, and hemostatic device removal time. The pooled odds ratio (OR), weighted mean difference (WMD), and standardized mean difference (SMD) with 95% confidence interval (95% CI) were calculated for dichotomous and continuous variables, respectively.

**Results:**

A total of 9,054 patients from 14 studies were included in the meta-analysis, and we found no significant difference in catheterization/puncture failure (OR = 1.94, 95CI [0.97, 3.86], *P*=0.06), hematoma (OR = 0.97, 95CI [0.55, 1.73], *P*=0.926), radial artery spasm (OR = 0.76, 95CI [0.43, 1.36], *P*=0.354), total procedure time (SMD = 0.23, 95CI [−0.21, 0.68], *P*=0.308), or radiation dose area product (WMD = 216.88 Gy/cm^2^, 95CI [−126.24, 560.00], *P*=0.215), but dTRA had a lower incidence of RAO (OR = 0.39, 95CI [0.23, 0.66], *P* < 0.001), shorter hemostatic device removal time (WMD = −66.62 min, 95CI [−76.68, −56.56], *P* < 0.001), longer access time (SMD = 0.32, 95CI [0.08, 0.56], *P*=0.008), and longer fluoroscopy time (SMD = 0.16, 95CI [−0.00, 0.33], *P*=0.05) than cTRA.

**Conclusion:**

Compared with the cTRA, the dTRA has a lower incidence of radial artery occlusion and shorter hemostatic device removal time, which is worthy of further evaluation in clinical practice.

## 1. Introduction

Compared with the transfemoral access, the transradial access is now widely used in coronary angiography and intervention, with the advantage of reducing the risk of bleeding, lowering the incidence of postoperative adverse events, and improving postoperative comfort [1]. However, due to the low release of nitric oxide, endothelial damage, and reduced blood flow caused by the insertion of the sheath and catheter, the conventional transradial access (cTRA) is more prone to complications such as radial artery spasm and radial artery occlusion (RAO) [2, 3], which can easily prolong the procedure time, choosing other arterial access, and increasing patient discomfort.

The distal transradial access (dTRA) in anatomical snuffbox is a new method proposed by Kiemeneij in 2017, which is safe and effective in performing coronary angiography and intervention to reduce hemostasis time [4]. Since the method was proposed, a meta-analysis [5] and several observational studies comparing the advantages and disadvantages of cTRA versus dTRA have emerged. Since the publication of the previous meta-analysis, some additional observational studies have been added. Therefore, this meta-analysis aimed to compare cTRA versus dTRA.

## 2. Materials and Methods

This work was conducted and reported according to Meta-analysis Of Observational Studies in Epidemiology (MOOSE) [6]. In addition, this meta-analysis has been registered with PROSPERO (registration number: CRD42020208776).

### 2.1. Search Strategy and Inclusion Criteria

We used Pubmed, Embase, and Cochrane Library databases to find studies of dTRA in anatomical snuffbox versus cTRA for coronary angiography and intervention, using the following search terms: “distal,” “snuffbox,” “snuff box,” “transradial,” “radial,” “coronary angiography,” and “Percutaneous Coronary Intervention.” The retrieval time was performed from the literature published until 5 January 2021, with no language restrictions. References in relevant articles were also accessed to find eligible studies. See online supplementary file 1 for detailed search strategies.

The studies included in this meta-analysis were required to meet the following criteria: (1) adults undergoing coronary angiography or intervention; (2) randomized controlled trials or observational studies of dTRA in anatomical snuffbox versus cTRA; (3) studies that included one of the following indicators: catheterization/puncture failure, hematoma, radial artery spasm, radial artery occlusion, access time, fluoroscopy time, radiation dose area product, total procedure time, and hemostatic device removal time; (5) case reports, conference abstracts, letters, reviews, and comments were excluded; and (6) the language of the studies was restricted to English.

### 2.2. Data Extraction and Quality Assessment

Two authors independently read the full text and extracted basic information about the eligible studies: first author, study type, country, publication year, sample size, basis characteristics of included patients, and indicators. For cohort studies, we used the nine-star Newcastle–Ottawa scale [7] to assess quality in terms of study population selection, comparability, and outcome. For randomized controlled trials, we used the Jadad scale [8] to evaluate quality in terms of the randomization method, blinding, loss of follow-up, and withdrawal, with a score ≤ 2 indicating low quality and a score ≥3 indicating high quality. Any differences were resolved by the third author.

### 2.3. Statistical Analysis

We used Stata version 14.0 for meta-analysis. The pooled odds ratio (OR) and its 95% confidence interval (95% CI) were calculated for dichotomous variables. The weighted mean difference (WMD) or the standardized mean difference (SMD) and its 95% CI were calculated for continuous variables. Heterogeneity among the studies was assessed with *I*^2^. If *I*^2^ > 50%, a random-effect model was used, and sensitivity analysis was performed by removing outlier studies. The Egger test was used to evaluate publication bias. A two-sided *P* < 0.05 was considered statistically different. If publication bias was present, effect sizes were recalculated using the trim and fill method.

## 3. Results

### 3.1. The Basic Characteristics of the Included Studies

**Figure 1:**
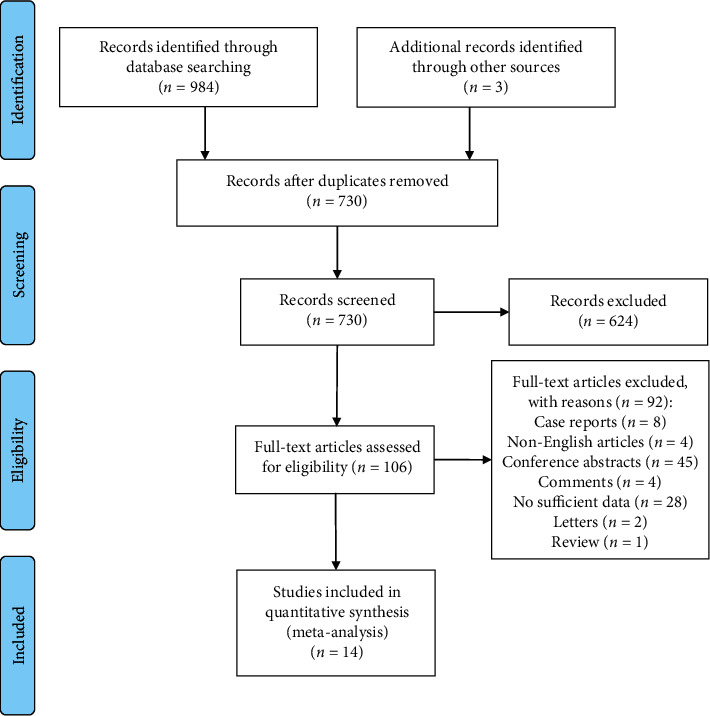
Flow chart of the search process.

The initial search identified 987 articles and 257 duplicate articles, 624 irrelevant articles were excluded based on the title and abstract, and 92 articles were excluded after viewing the full text. Finally, 14 studies met the inclusion criteria.  Figure 1 shows the detailed search process.

Table 1 presents basic information of the 14 eligible studies, including 2 randomized controlled trials and 12 cohort studies (including 1 retrospective study and 11 prospective studies), involving a total of 9,054 patients with sample sizes ranging from 41 to 5,874. The included studies were all of high quality.

### 3.2. Meta-Analysis of Indicators

#### 3.2.1. Catheterization/Puncture Failure

**Figure 2:**
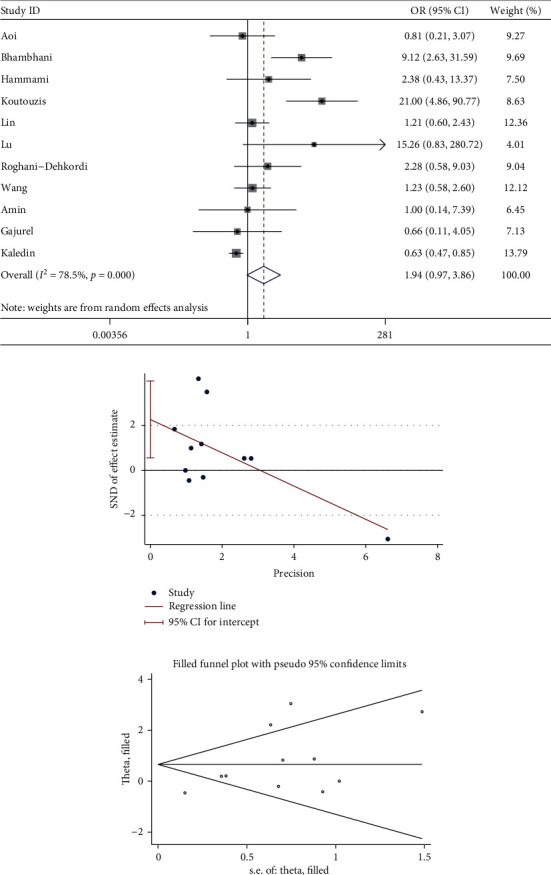
(a) Forest plot of catheterization/puncture failure for dTRA compared to cTRA. (b) Egger test was used to quantitatively assess publication bias in catheterization/puncture failure (*P*=0.015). (c) Trim and fill funnel plot showed that no new studies were added.

11 studies described catheterization/puncture failure. The meta-analysis showed similar catheterization/puncture failure between dTRA and cTRA, with no statistical significance (OR = 1.94, 95CI [0.97, 3.86], *P*=0.06; *I*^2^ = 78.5%, *P* < 0.001). We conducted a sensitivity analysis by removing outlier studies, heterogeneity was significantly reduced after excluding the studies by Bhambhani [11] and Koutouzis [17], and there was still no statistical difference (OR = 1.06, 95CI [0.69, 1.64], *P*=0.788; *I*^2^ = 38.2%, *P*=0.114). The Egger test suggested the existence of publication bias among the studies (*P*=0.015), so we used the trim and fill method to recalculate the effect size: the number of studies did not change, and there was still no statistical difference between them (OR = 1.927, 95CI [0.981, 3.785]), suggesting that the result was robust (see Figure 2).

#### 3.2.2. Hematoma

**Figure 3:**
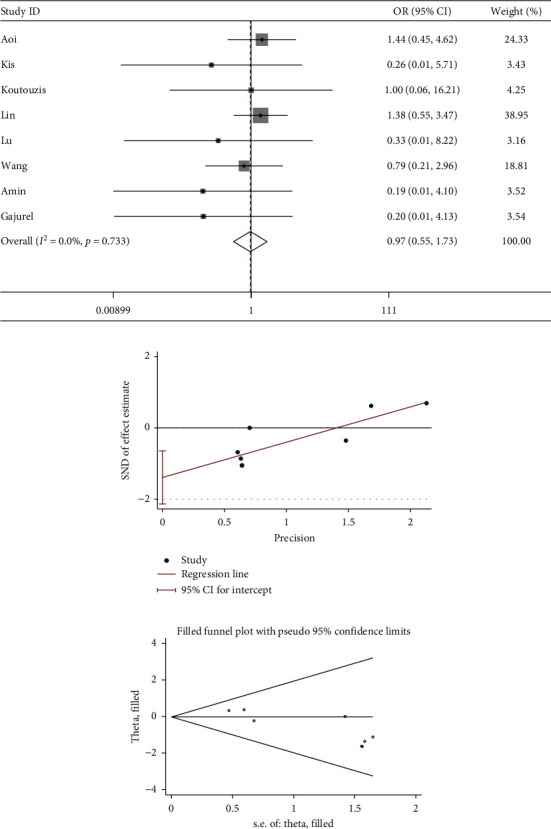
(a) Forest plot of hematoma for dTRA compared to cTRA. (b) Egger test was used to quantitatively assess publication bias in hematoma (*P*=0.004). (c) Trim and fill funnel plot showed that no new studies were added.

Eight studies described hematoma. The meta-analysis showed the incidence of hematoma was similar between dTRA and cTRA, with no statistical significance (OR = 0.97, 95CI [0.55, 1.73], *P*=0.926; *I*^2^ = 0.0%, *P*=0.733). The Egger test suggested the existence of publication bias among the studies (*P*=0.004), so we used the trim and fill method to recalculate the effect size: the number of studies did not change, and there was still no statistical difference (OR = 0.973, 95CI [0.548, 1.728]), suggesting that the result was robust (see Figure 3).

#### 3.2.3. Radial Artery Spasm

**Figure 4:**
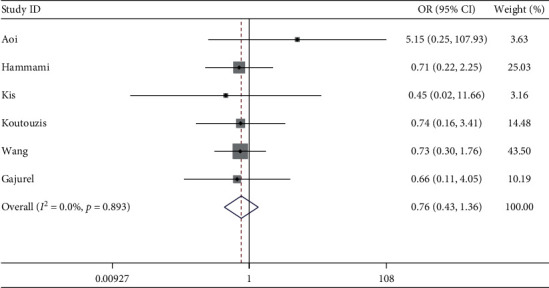
Forest plot of radial artery spasm for dTRA compared to cTRA.

Six studies described radial artery spasm. The meta-analysis showed the incidence of radial artery spasm was similar between dTRA and cTRA, with no statistical significance (OR = 0.76, 95CI [0.43, 1.36], *P*=0.354; *I*^2^ = 0.0%, *P*=0.893) (see Figure 4). The Egger test showed no publication bias among the studies (*P*=0.437).

#### 3.2.4. Radial Artery Occlusion (RAO)

**Figure 5:**
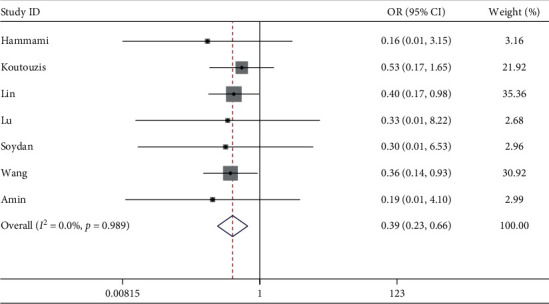
Forest plot of radial artery occlusion for dTRA compared to cTRA.

Seven studies described RAO. The meta-analysis showed compared with cTRA, the incidence of RAO was lower in dTRA, with the statistical difference between studies (OR = 0.39, 95CI [0.23, 0.66], *P* < 0.001; *I*^2^ = 0.0%, *P*=0.989) (see Figure 5). The Egger test showed no publication bias among the studies (*P*=0.115).

#### 3.2.5. Access Time

**Figure 6:**
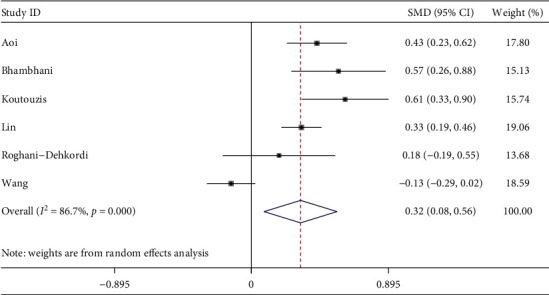
Forest plot of access time for dTRA compared to cTRA.

Six studies described access time. The meta-analysis showed compared with cTRA, the access time was longer in dTRA, with the statistical difference between studies (SMD = 0.32, 95CI [0.08, 0.56], *P*=0.008; *I*^2^ = 86.7%, *P* < 0.001) (see Figure 6). We conducted a sensitivity analysis by removing the outlier study, heterogeneity was significantly reduced after excluding the study by Wang [22], and there was still a statistical difference (SMD = 0.41, 95CI [0.29, 0.54], *P* < 0.001; *I*^2^ = 32.5%, *P*=0.205). The Egger test showed no publication bias among the studies (*P*=0.463).

#### 3.2.6. Fluoroscopy Time

**Figure 7:**
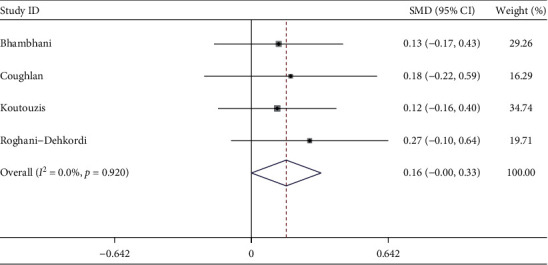
Forest plot of fluoroscopy time for dTRA compared to cTRA.

Four studies described fluoroscopy time. The meta-analysis showed compared with cTRA, the fluoroscopy time was longer in dTRA, with the statistical difference between studies (SMD = 0.16, 95CI [−0.00, 0.33], *P*=0.05; *I*^2^ = 0.0%, *P*=0.920) (see Figure 7). The Egger test showed no publication bias among the studies (*P*=0.246).

#### 3.2.7. Radiation Dose Area Product (DAP)

**Figure 8:**
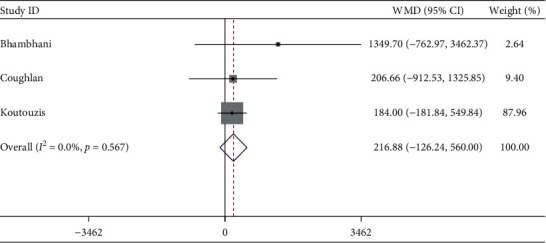
Forest plot of radiation dose area product for dTRA compared to cTRA.

Three studies described DAP. The meta-analysis showed the DAP was similar between dTRA and cTRA, with no statistical significance (WMD = 216.88 Gy/cm^2^, 95CI [−126.24, 560.00], *P*=0.215; *I*^2^ = 0.0%, *P*=0.567) (see Figure 8). The Egger test showed no publication bias among the studies (*P*=0.414).

#### 3.2.8. Total Procedure Time

**Figure 9:**
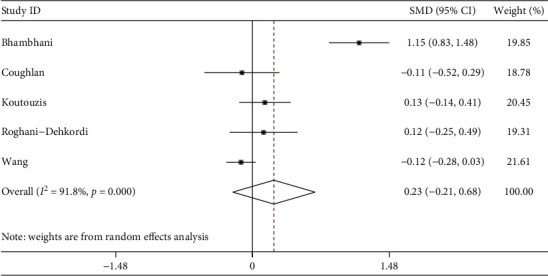
Forest plot of total procedure time for dTRA compared to cTRA.

Five studies described total procedure time. The meta-analysis showed the total procedure time was similar between dTRA and cTRA, with no statistical significance (SMD = 0.23, 95CI [−0.21, 0.68], *P*=0.308; *I*^2^ = 91.8%, *P* < 0.001) (see Figure 9). We conducted a sensitivity analysis by removing the outlier study, heterogeneity was significantly reduced after excluding the study by Bhambhani [11], and there was still no statistical difference (SMD = −0.03, 95CI [−0.17, 0.11], *P*=0.642; *I*^2^ = 13%, *P*=0.328). The Egger test showed no publication bias among the studies (*P*=0.398).

#### 3.2.9. Hemostatic Device Removal Time

**Figure 10:**
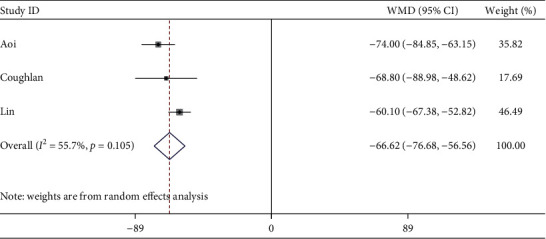
Forest plot of hemostatic device removal time for dTRA compared to cTRA.

Three studies described hemostatic device removal time. The meta-analysis showed compared with cTRA, the hemostatic device removal time was shorter in dTRA, with the statistical difference between studies (WMD = −66.62 min, 95CI [−76.68, −56.56], *P* < 0.001; *I*^2^ = 55.7%, *P*=0.105) (see Figure 10). We conducted a sensitivity analysis by removing the outlier study, heterogeneity was significantly reduced after excluding the study by Lin [18], and there was still a statistical difference (WMD = −72.83 min, 95CI [−82.39, −63.27], *P* < 0.001; *I*^2^ = 0.0%, *P*=0.656). The Egger test showed no publication bias among the studies (*P*=0.588).

## 4. Discussion

This work aimed to evaluate the differences between dTRA in anatomical snuffbox versus cTRA. Our meta-analysis of the 14 included studies found no significant differences in catheterization/puncture failure, hematoma, radial artery spasm, total procedure time, or radiation dose area product, but dTRA had a lower incidence of RAO, shorter hemostatic device removal time, and longer access time and fluoroscopy time than cTRA.

The anatomical snuffbox is a triangular depression of the tip towards the thumb when the thumb is fully extended. The radial artery in the anatomical snuffbox is superficially located and can be palpated [23]. The vascular diameter of the distal radial artery in the anatomical snuffbox is usually smaller than that of the radial artery at the wrist, with a ratio of about 0.8–0.9 [24], while the diameter of the distal radial artery in men is larger than that in women [25], which means that successful distal radial artery catheterization/puncture seems to be more challenging. The overall catheterization/puncture failure of dTRA included in this meta-analysis was higher than that of cTRA, but there was no statistically significant difference (4.3% VS 3.8%, *P* > 0.05), and the access time was prolonged in dTRA, but did not affect total procedure time. Bhambhani [11] performed distal radial artery cannulation in 100 patients and found access time in dTRA was progressively reduced, from 5.89 minutes in the first 25 cases to 2.47 minutes in the last 25 cases. Also, Lee [26] found that puncture time stabilized after approximately 150 distal radial artery punctures had been performed. Therefore, the learning curve must be overcome to master the dTRA.

The anatomical snuffbox is surrounded by soft tissue and has a bony base consisting of the scaphoid bone and trapezium bone, which can be easily compressed to hemostasis, and the complications of bleeding and hematomas are uncommon [23, 27]. The radial artery divides into the superficial palmar branch near the level of the AS to form the superficial palmar arch with the terminal ulnar artery, and the terminal of the radial artery forms the deep palmar arch at the distal end of the anatomical snuffbox with the deep palmar branch of the ulnar artery. There are abundant collateral anastomoses between the two arches. Even if the distal radial artery is occluded, antegrade flow is maintained and the risk of retrograde thrombosis is reduced [23, 28, 29]. This meta-analysis found that compared with cTRA, hemostatic device removal time was shorter in dTRA (WMD = −66.62 min, 95CI [−76.68, −56.56], *P* < 0.001), the incidence of radial artery occlusion was lower in dTRA (4.6% vs 1.7%, *P* < 0.001), and the incidence of hematoma (2.1% vs 1.8%, *P*=0.926) and radial artery spasm (3.4% vs 2.6%, *P*=0.354) was similar in dTRA, suggesting that the dTRA was more comfortable and safer for patients.

When performing the left dTRA, the patient can naturally place the left hand at the level of the right groin, allowing the operator on the right side of the patient to not bend over. This allows the operator to keep away from the source of radiation and avoid high radiation doses and solve the problem of postoperative restriction of the right hand, which is extremely comfortable for right-handed patients [4]. This meta-analysis found no significant difference in radiation dose area product between dTRA and cTRA (*P*=0.215), but the fluoroscopy time in dTRA was prolonged (*P*=0.05). As *P*=0.05 represents the critical *P* value, which is at the borderline of statistical significance, this result should be interpreted with more caution and needs to be further demonstrated in large-sample studies.

Despite the advantages of shorter hemostatic device removal time and lower incidence of RAO, the limitations of this novel access should be noted: smaller vascular diameter limits the use of large bore sheaths, and longer access time may delay coronary revascularization, especially in cases of myocardial infarction.

There are some limitations in this meta-analysis. First, publication bias was present in the catheterization/puncture failure and hematoma. Positive results tended to be more likely to be published than negative results, small sample publication bias, and/or lack of studies with opposite results could explain this publication bias. Despite the presence of publication bias, the results were reliable after using the trim and fill method to recalculate the effect sizes. In addition, heterogeneity existed in some indicators with the presence of critical *P* value in fluoroscopy time, which may have been caused by differences in operator experience and inclusion groups. Therefore, a large sample size of randomized controlled trials is still needed for further assessment. Finally, due to the lack of sufficient data, we did not analyze the differences in access time and/or radiation exposure between the left and the right arm, which still need further evaluation.

## 5. Conclusions

Compared with the cTRA, the dTRA has a longer access time and fluoroscopy time, but this does not affect total procedure time or radiation dose area product. Also, the dTRA has a lower incidence of radial artery occlusion and shorter hemostatic device removal time, which is worthy of further evaluation in clinical practice.

## Figures and Tables

**Table 1:** Basic information of the included studies.

Author	Year	Country	Study type	Sample	Male (%)	Overall age	Hypertension (%)	Diabetes mellitus (%)	Smoking (%)	Hyperlipidemia (%)	BMI	Indicators	NOS score	Jadad score
Aoi [9]	2019	USA	Retrospective	408 (202 vs. 206)	64.9 vs.62.6	69.2 vs.68.8	85.6 vs.95.1	37.6 vs.45.6	33.3 vs.25.2	83.7 vs.91.7	26.5 vs.27.1	1, 2, 3, 5, 9	7 (3/2/2)	—
Amin [10]	2017	Bangladesh	Prospective	100 (50 vs.50)	—	—	—	—	—	—	—	1, 2, 4	7 (4/0/3)	—
Bhambhani [11]	2020	India	Prospective	200 (100 vs.100)	84 vs.73	54.6 vs.54.9	59 vs.50	40 vs.35	39 vs.32	—	—	1, 5, 6, 7, 8	9 (4/2/3)	—
Coughlan [12]	2018	Ireland	Prospective	94 (47 vs.47)	83 vs.74.5	61 vs.61.8	—	—	—	—	—	6, 7, 8, 9	9 (4/2/3)	—
Gajurel [13]	2018	Nepal	Prospective	164 (82 vs.82)	58.5 vs.53.6	57.7 vs.57.2	35.3 vs.29.2	25.6 vs.18.2	43.9 vs.32.9	20.7 vs.12.1	—	1, 2, 3	9 (4/2/3)	—
Hammami [14]	2021	Tunisia	Prospective	177 (82 vs. 95)	62 vs.70	59.23 vs.60.38	40 vs.44	45 vs.40	41.5 vs.42	—	—	1, 3, 4	9 (4/2/3)	—
Kis [15]	2020	Turkey	Prospective	41 (17 vs.24)	70.6 vs.70.8	—	70.6 vs.79.2	47.1 vs.33.3	35.3 vs.29.2	41.2 vs.37.5	—	2, 3	9 (4/2/3)	—
Kaledin [16]	2017	Russia	Prospective	5874 (2775 vs.3099)	—	—	—	—	—	—	—	1	7 (4/0/3)	—
Koutouzis [17]	2019	Greece	Randomized	200 (100 vs.100)	74 vs.77	63.8 vs.62.8	73 vs.63	27 vs.28	35 vs.28	71 vs.59	28.6 vs.29	1, 2, 3, 4, 5, 6, 7, 8	—	3 (2/0/1)
Lin [18]	2020	China	Randomized	900 (450 vs.450)	45.56 vs.50	55.28 vs.58.81	24.89 vs.25.11	10.67 vs.12.44	27.56 vs.22.44	—	24.06 vs.24.36	1, 2, 4, 5, 9	—	3 (2/0/1)
Lu [19]	2020	China	Prospective	80 (40 vs.40)	57.5 vs.62.5	54.3 vs.56.4	—	15 vs.17.5	—	—	—	1, 2, 4	8 (4/2/2)	—
Roghani-Dehkordi [20]	2020	Iran	Prospective	126 (70 vs.56)	62.8 vs.64.3	55.1 vs.56.5	57.1 vs.67.8	74.2 vs.80.3	67.1 vs.78.6	31.4 vs.28.6	28.4 vs.27.2	1, 5, 6, 8	9 (4/2/3)	—
Soydan [21]	2021	Turkey	Prospective	70 (27 vs.43)	63 vs.72.1	—	77.8 vs.76.7	40.7 vs.39.5	33.3 vs.27.9	48.1 vs.30.2	—	4	9 (4/2/3)	—
Wang [22]	2020	China	Prospective	620 (312 vs.308)	51.3 vs.51	50.1 vs.51.2	60.6 vs.56.5	31.4 vs.28.2	56.4 vs.54.5	44.2 vs.41.9	—	1, 2, 3, 4, 5, 8	8 (4/2/2)	—

1- Catheterization/puncture failure; 2- hematoma; 3- radial artery spasm; 4- radial artery occlusion; 5- access time; 6- fluoroscopy time; 7- radiation dose area product; 8- total procedure time; 9- hemostatic device removal time.

## Data Availability

The datasets used and/or analysed during the current study are available from the corresponding author on reasonable request.

## Supplementary Materials

Supplementary Materials Online supplementary file 1: search strategies.
