# Supplementary material for: Distal Transradial Access in Anatomical Snuffbox for Coronary Angiography and Intervention: An Updated Meta-Analysis
Source: J Interv Cardiol. 2021 Jul 22;2021:7099044. doi: 10.1155/2021/7099044 (PMC8321763; doi:10.1155/2021/7099044)
Supplement: Supplementary Materials — Online supplementary file 1: search strategies. [file 7099044.f1.docx]

**Search strategies**

**Pubmed**

1.(("distal"[Title/Abstract]) OR ("snuffbox"[Title/Abstract])) OR ("snuff box"[Title/Abstract])

2.("radial"[Title/Abstract]) OR ("transradial"[Title/Abstract])

3.((((((("Coronary Angiography"[Mesh]) OR ("coronary angiography"[Title/Abstract])) OR ("coronary angiographies"[Title/Abstract])) OR ("Percutaneous Coronary Intervention"[Mesh])) OR ("coronary intervention"[Title/Abstract])) OR ("coronary interventions"[Title/Abstract])) OR ("coronary revascularization"[Title/Abstract])) OR ("coronary revascularizations"[Title/Abstract])

4. 1 AND 2 AND 3

**Embase**

#1. 'distal':ab,ti OR 'snuffbox':ab,ti OR 'snuff box':ab,ti

#2. 'transradial':ab,ti OR 'radial':ab,ti

#3. 'coronary angiography'/exp

#4. 'percutaneous coronary intervention'/exp

#5. 'coronary angiography':ab,ti OR 'coronary angiographies':ab,ti OR 'coronary intervention':ab,ti OR 'coronary interventions':ab,ti OR 'coronary revascularization':ab,ti OR 'coronary revascularizations':ab,ti

#6. #3 OR #4 OR #5

#7. #1 AND #2 AND #6

**Cochrane Library**

#1 (“distal”):ti,ab,kw OR (“snuffbox”):ti,ab,kw OR (“snuff box”):ti,ab,kw (Word variations have been searched)

#2 (“transradial”):ti,ab,kw OR (“radial”):ti,ab,kw (Word variations have been searched)

#3 MeSH descriptor: [Coronary Angiography] explode all trees

#4 MeSH descriptor: [Percutaneous Coronary Intervention] explode all trees

#5 (“coronary angiography”):ti,ab,kw OR (“coronary angiographies”):ti,ab,kw (Word variations have been searched)

#6 (“coronary intervention”):ti,ab,kw OR (“coronary interventions”):ti,ab,kw OR (“coronary revascularization”):ti,ab,kw OR (“coronary revascularizations”):ti,ab,kw (Word variations have been searched)

#7 #3 OR #4 OR #5 OR #6

#8 #1 AND #2 AND #7
